# Regional differences in winter diets of bobcats in their northern range

**DOI:** 10.1002/ece3.4576

**Published:** 2018-10-09

**Authors:** Roberta K. Newbury, Karen E. Hodges

**Affiliations:** ^1^ Department of Biology University of British Columbia Okanagan Kelowna British Columbia Canada

**Keywords:** bobcat, diet, Montana, red squirrel, snowshoe hare, winter

## Abstract

When generalist predators have wide geographic ranges, diets may differ dramatically, largely as a result of differing prey communities. Bobcats (*Lynx rufus*) are widely distributed across southern North America, with their northern range edge occurring in southern Canada and in the northern US states. Within this northern range, bobcats are exposed to cold and snowy winters and a limited number of prey species, conditions that are atypical for most of the range of bobcats. We examined winter diets of bobcats in high elevation and very snowy forests in northwest Montana to determine how these generalist predators managed in these harsh conditions in comparison with elsewhere in the northern range. Bobcats consumed five major prey types: Red squirrels (*Tamiasciurus hudsonicus*) and Cricetid rodents comprised >78% of the dietary biomass, whereas the larger snowshoe hares (*Lepus americanus*), deer (*Odocoileus* spp.), and grouse were consumed much less often. The standardized niche breadth of bobcat diets was 0.29; bobcats from across the northern range also routinely ate multiple prey species, although Eastern bobcats appear to consume more lagomorphs than do Western bobcats. These results indicate that bobcats remain generalists in difficult winter conditions while preying primarily on small‐bodied prey, although bobcats have highly variable diets across their northern range.

## 1 INTRODUCTION

Generalist predator species often display high behavioral plasticity (Tuomainen & Candolin, 2011), and part of this flexibility is manifested in switching prey types when a given prey species becomes more numerous. This ability to use many prey types also allows generalists to use many different regions with varying prey bases, which can lead to a broad geographic distribution. Although a generalist may engage in facultative specialization in response to locally abundant or valuable resources, the plastic behavior of a generalist predator still allows them to use other prey species (Malo, Lozano, Huertas, & Virgós, 2004; Roth, Marshall, Murray, Nickerson, & Steury, 2007), often seamlessly switching prey types without the delay in prey switching seen in specialist species (O'Donoghue, Boutin, Krebs, Murray, & Hofer, 1998).

Bobcats (*Lynx rufus*) are common North American cats that use many habitat types and prey species (Fuller, Berg, & Kuehn, 1985; Litvaitis, Sherburne, & Bissonette, 1986; McCord & Cardoza, 1982). Bobcats are widely distributed throughout the United States, but are less common in southern Canada and northern Mexico (Anderson, 1987). The northern range margin of bobcats in British Columbia, Canada, occurs at ~53.5–54.5°N (near Highway 16; Gooliaff, Weir, & Hodges, 2018), and this range edge has been stable for the last eight decades (Gooliaff & Hodges, 2018). In these northwestern subboreal and boreal forests, few prey species are available in winter compared to the southern part of the range. Further, throughout their northern range, bobcats overlap with a specialist congener, Canada lynx (*Lynx canadensis*), which relies on boreal forest for habitat and snowshoe hares (*Lepus americanus*) for prey (Mowat, Poole, & O'Donoghue, 2000; Roth et al., 2007). Lynx have morphological adaptations for snowy winters, including large feet that reduce foot‐loading and long hind legs that facilitate travel, hunting, and capture of hares in deep, soft snow (Murray & Boutin, 1991). In contrast, bobcats do not face severe winters throughout most of their geographic range. Snow depth negatively influences bobcat movements (McCord, 1974) and habitat use (Bailey, 1974). Bobcats have small feet that sink into soft snow, putting bobcats at an energetic disadvantage in environments with deep snow; bobcats expend larger amounts of energy than do Canada lynx in locations with cold, snowy winters (Buskirk, Ruggiero, & Krebs, 2000; Parker & Smith, 1983).

Given that the northern range edge for bobcats occurs in a region with limited winter prey and snow conditions that would seem to favor lynx over bobcats, the query becomes how bobcats manage the challenges of limited prey and the presence of a specialist congener. In these winter forests, the ~1,400‐g hares offer substantially more calories than do the ~200‐g red squirrels (*Tamiasciurus hudsonicus*) and <40‐g small mammals that are available. There are three basic ways the dietary flexibility of bobcats could manifest in this setting: (a) via bobcats “becoming lynx‐like” by acting as facultative specialists and preying primarily on the energetically rich snowshoe hares; (b) by eating a suite of species, including hares; or (c) by focusing on prey other than hares. Previous evidence is mixed; bobcats in Eastern North America consumed >50% hares in winter (Litvaitis & Harrison, 1989; Litvaitis, Clark, & Hunt, 1986; Matlack & Evans, 1992; Pollack, 1951), whereas bobcats in Idaho consumed only 1.5% hares (Koehler & Hornocker, 1989).

We thus have two research objectives. First, we characterize bobcat diets to assess how specialized their winter diets are in a region of Montana that is higher elevation and much snowier than study areas used in previous work on bobcat diets in their northern range. Second, we compare the diets of these montane bobcats in northwestern Montana (hereafter “Montana bobcats”) to bobcats from elsewhere in the northern range, to assess how flexible bobcats are in their diets across areas that experience prolonged snowy winters. For this objective, we determined dietary niche breadths of northern bobcats after a thorough literature search for data on bobcat diets in northern latitudes. For both objectives, we are particularly interested in how prevalent snowshoe hares are in bobcat diets, as these prey do not occur in the southern range of bobcats and because hares are the primary prey of Canada lynx.

## 2 MATERIALS AND METHODS

Our study area was the Tally Lake Ranger District of the Flathead National Forest, northwestern Montana, USA (48°30′0″N, 114°45′0″W), located in the center of the Salish Range. The Salish Mountains (48°12′N, 114°48′W) encompass 10,684 km^2^, with >30 peaks over 1,828 m, of which 10 peaks were located in our study area. TLRD encompasses 1,137 km^2^, with elevations ranging from 945 to 2,008 m. Annual temperatures range from −42 to 38°C and mean annual precipitation is 58 cm at 975 m in Olney, Montana, on the northeast edge of the TLRD (NOAA, 2017). Winter temperatures range from −42 to 7°C, and annual snowfall typically exceeds 300 cm at elevations >1,300 m and can exceed 700 cm at elevations >2,000 m (NOAA, 2017).

Forested areas were dominated by moist coniferous forests composed of Western larch (*Larix occidentalis*), lodgepole pine (*Pinus contorta*), Douglas fir (*Pseudotsuga menziesii*), subalpine fir (*Abies lasiocarpa*), and Engelmann spruce (*Picea engelmannii*). Lodgepole pine forests formed 30% of the landscape, and an additional 30% was formed by Douglas fir/larch associations. Subalpine fir forests constituted 20% of the area (Flathead National Forest, 2006). The remaining area was composed of Ponderosa pine (*Pinus ponderosa*), Western Red Cedar (*Thuja plicata*)/Western hemlock (*Tsuga heterophylla*), grand fir (*Abies grandis*), and whitebark pine (*Pinus albicaulis*)/subalpine larch (*Larix lyallii*) communities.

During winter, snowshoe hares, red squirrels, grouse (*Falcipennis canadensis* and *Bonasa umbellus*), bushy‐tailed woodrats (*Neotoma cinerea*), and a variety of small mammals (mice and vole subfamilies Neotominae and Arvicolinae, respectively) are possible food sources. Deer (*Odocoileus virginianus* and *Odocoileus hemionus*) are present but uncommon on the higher elevations of the study area in winter. Carrion (including deer, elk *Cervus canadensis*, and moose *Alces alces*) may also be available to bobcats.

### 2.1 Sample collection

**Figure 1:**
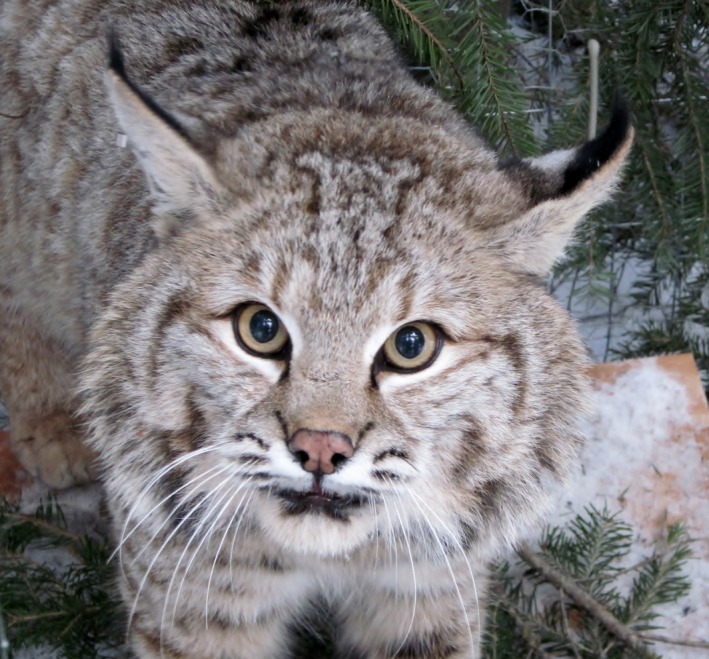
An adult male bobcat (*Lynx rufus pallescens*), M1, that was captured and radio‐collared as part of this study on the Tally Lake Ranger District, Flathead National Forest, northwest Montana. M1 weighed ~15 kg when collared on 12 December 2009. In this photograph, M1 was recaptured on 25 January 2010 and released without handling

Bobcat scats were collected throughout the study area during winter (December–February, 2009–2011) when encountered along snowmobile tracks or while backtracking a bobcat. Appearance of the scat and the presence of bobcat tracks were used to confirm the scat was from a bobcat. Scats were also collected from live‐trapped bobcats (Figure 1) (Newbury, 2013). Scats collected from traps were assumed to be from the bobcat's meal prior to ingesting trap bait (deer), and indeed, no scats contained deer. Any fur from trap bait that was frozen or stuck to the outside of scats was removed. Live‐trapping adhered to strict protocols for trapping and handling and permits from Montana State Fish, Wildlife, and Parks (2009‐059, 2010‐002, 2011‐003), and the University of British Columbia's Animal Care Committee (A07‐0676‐R001); our work adheres to the guidelines of the American Society of Mammalogists (Sikes & the Animal Care & Use Committee of the American Society of Mammalogists, 2016).

Bobcat carcasses were collected by voluntary donation from licensed local fur trappers. All kill‐trapped bobcats came from the study area and the Salish Mountain range immediately surrounding this area. The trapping season officially runs from 1 December–15 February; however, all carcasses were collected in December, as the bobcat quota was filled by the end of December. We collected 30 carcasses in 2009 and 17 carcasses in 2010. Necropsies were conducted at the Montana Fish, Wildlife, and Parks Wildlife Laboratory in Bozeman, Montana, and the Philip L. Wright Zoological Museum preparatory laboratory at the University of Montana in Missoula, Montana. Stomachs were opened and all contents removed. Colon contents were collected from the section of large intestine within 15.25 cm of the rectum, such that colon samples were basically the same as scat samples; stomach and colon samples were assumed to represent different meals. We retained both samples in subsequent analyses, so 21 carcasses provided two samples, 16 carcasses had colon samples only, and four had stomach samples only. Similarly, we do not know whether all scats were from separate individuals. We combined stomach, colon, and scat samples in our analysis. Stomach samples are less digested than colon samples, so feathers, fur, and bones were often more identifiable than in scats; however, in both stomach and scat/colon samples, we could not always separate small mammals to species or genus (deer, grouse, squirrel, and hare remained identifiable). These samples thus provide comparable information, and we do not think there is a bias from combining sample types. Samples were stored at −23°C until 24–48 hr prior to analysis, when they were thawed at room temperature.

### 2.2 Sample analysis and prey identification

All scat and colon samples were oven‐dried until sample mass remained constant. Sample contents were analyzed following Reynolds and Aebischer (1991). Dry mass of each scat or colon sample was recorded; then, samples were broken down in water and rinsed through a 0.5‐mm sieve to separate microscopic from macroscopic fragments. After thawing, stomach samples were immediately rinsed through a 0.5‐mm sieve (Litvaitis, Stevens, & Mautz, 1984). The 0.5‐mm mesh captured even the smallest rodent bones and teeth. Each sample was sorted into categories such as fur, bone, feather, and incidental ingestion (e.g. pine needles) and then air‐dried prior to identifying species.

Prey items were identified to species by using diagnostic hair, teeth, and bones. Bones and fur present in samples were compared to specimens in the Philip L. Wright Zoological Museum for species confirmation. When no diagnostic teeth or bones were present, hairs were identified by using a compound microscope, reference hairs, and a key to mammalian guard hairs (Moore, Spence, & Dugnolle, 1974). This approach was often necessary for mice and voles, although sometimes we were able to identify only to subfamily or family for rodents because of severe degradation of hair and bone in samples.

We excluded probable trap bait in two ways. First, deer tracks were rarely located on our study site in winter, but we used road‐killed deer to bait live traps. We found no deer in scats collected from live‐trapped animals or from scats found along tracks and roads in the trapping area. Second, to account for trap bait in stomach and colon samples from bobcat carcasses, we sent surveys to trappers who had turned in bobcat carcasses. When we received a trapper's response (~50%), we removed that bait type from prey remains in the gut. None of the trappers who responded had used red squirrel or snowshoe hare as bait. We also excluded items such as domestic chicken that were very likely to be bait. However, we did not exclude deer from samples where the trapper did not respond, because for some samples for which a trapper did respond, deer fur/meat was contained in the sample, but the trapper had not used deer as bait.

After prey species were identified, the volume of each sample composed of that species was visually estimated following Reynolds and Aebischer (1991). Most samples (83%) were composed of one prey species. We were not able to quantify the number of individuals in each sample, given the degraded quality of bone and fur. This decision may underestimate individual Cricetid rodents consumed, but is unlikely to underestimate the number of larger prey.

### 2.3 Statistical analyses

We calculated absolute frequency of occurrence (AFO) of each prey species found (number of occurrences of a given prey type/total number of samples; Wright, 2010), and relative frequency of occurrence (RFO; number of occurrences of a given prey type/total number of prey species occurrences). RFO accounts for more than one prey type being found in some samples (Ackerman, Lindzey, & Hemker, 1984).

We estimated the biomass consumed of each prey species from Baker's , Warren, and James (1993) regression equation for bobcats that relates dry mass of each prey type in the scat to the fresh consumed biomass. Following Baker et al.’s (1993) regression equation *y* = 16.63 + 4.09*x*, where *x* is the average mass of each prey type (Table 1) and *y* is the biomass consumed, we calculated conversion factors for each prey type except deer. For deer, we used the empirical results from Baker et al. (1993), that is, a conversion factor of 27.0. Dry masses per prey type in stomach samples were not determined because stomach samples were not dried prior to analysis. To incorporate stomach samples into biomass estimates, we used the average dry weight of each prey type from the scat and colon samples; for example, each stomach sample that contained deer was assigned a value of 5.5 g of deer. We summed the total dry mass per prey type in our samples, multiplied by the conversion factor, and then divided by total mass summed across all prey types to determine percent biomass consumed of each prey type (Baker et al., 1993).

**Table 1:** Prey species potentially present in the Salish Range and Tally Lake Ranger District in winter, based upon Foresman (2001)

Prey	Common name	Average body mass (kg)
Cervidae		60.0[Link]
*Odocoileus hemionus*	Mule deer	
*Odocoileus virginianus*	Whitetail deer	
Leporidae		
*Lepus americanus*	Snowshoe hare	1.4
Sciuridae		
*Tamiasciurus hudsonicus*	American red squirrel	0.195
Tetraoninae		0.539
*Bonasa umbellus*	Ruffed grouse	
*Falcipennis canadensis*	Spruce grouse	
Cricetidae		0.038[Link]
Arvicolinae[Link], [Link]		
*Microtus longicaudus*	Long‐tailed vole	
*Microtus montanus*	Montane vole	
*Microtus pennsylvanicus*	Meadow vole	
*Microtus richardsoni*	Water vole	
*Myodes gapperi*	Southern red‐backed vole	
*Ondatra zibethicus*	Muskrat	1.136
*Phenacomys intermedius*	Heather vole	
*Synaptomys borealis*	Northern bog lemming	
Neotominae[Link]		
*Neotoma cinerea*	Bushy‐tailed woodrat	0.336
*Onychomys leucogaster*	Northern grasshopper mouse	
*Peromyscus leucopus*	White‐footed mouse	
*Peromyscus maniculatus*	Deer mouse	
*Reithrodontomys megalotis*	Western harvest mouse	

We used median mass for deer to account for differences between sex and age classes; adult deer have higher average biomass than shown here.

This average mass was used for all Cricetidae, except muskrats and woodrats.

*Myodes gapperi* and *Microtus* spp. are most common on the study site. *Ondatra zibethicus* are also common in the area, and were easy to identify in remains compared to the smaller arvicolids.

*Neotoma cinerea* and *Peromyscus maniculatus* were most common on the study area and were easy to distinguish from one another in remains.

We then compared diets of bobcats in our study area to diets of bobcats from similar northern latitudes but across a wide longitudinal gradient. We searched Web of Science and the ProQuest database of theses and dissertations for bobcat winter dietary research in the northern range. We lumped the data into Eastern and Western states/provinces, because finer geographic subdivision resulted in very uneven sample sizes; some studies also lumped data from several states. Although we present data from midwestern populations, we do not compare these analytically because of the low sample size of studies. In studies from which absolute frequency of occurrence data could be extracted, we grouped diet into six categories: Cervidae, Lagomorpha, Sciuridae, Rodentia, Aves, and Other. We then used a *G* test for independence with a significance value of *p* < 0.05 to compare average proportions of each prey category reported in studies of winter bobcat diets.

### 2.4 Dietary niche breadth and overlap among bobcat populations

Winter niche breadth for Montana bobcats and other bobcats in northern latitudes was calculated from AFO in Levins (1968) measure of niche breadth $B=1/\sum \;p_{j}^{2}$, where *B* = niche breadth and *p_j_ *= fraction of items in the diet that are of food category *j*. We converted it to a standardized dietary breadth on a scale of 0–1 following Hurlbert's (1978) measure: BA* *= (*B* − 1)/(*n* − 1), where BA* *= standardized niche breadth, *B* = niche breadth, and *n* = number of possible resources (Krebs, 1998).

We examined dietary overlap among northern bobcat populations to see whether bobcat diets differed regionally despite all of our comparisons occurring in areas where bobcats experience snowy winters (in contrast to the southern United States and northern Mexico) and a similar prey base (deer, hares, squirrels, grouse, and small mammals were the main prey available in winter in the regions we compared). Dietary niche overlap for bobcat populations in Western and Eastern regions was calculated in EcoSim Professional v1.2d (Entsminger, 2014) using Pianka's (1974) index *α* = ∑*p_i_q_i_*/($\sum p_{i}^{2}\sum q_{i}^{2}$)^1/2^, where p_i_ is the proportion of prey type *i* in the diet of the first group and q_i_ is the proportion of the same prey type in the diet of the second group. The index ranges from 0 to 1, from no overlap to complete overlap. We ran 1,000 randomized simulations within EcoSim to determine whether the probability of observed overlaps was greater or less than expected by chance.

## 3 RESULTS

In northwestern Montana, bobcats consumed snowshoe hares as 17.9% (absolute frequency of occurrence) of their winter diet, while red squirrels and Cricetid rodents composed 48.7% and 34.6% of samples, respectively (Table 2). Squirrels were detected in 38 samples, other rodents in 27 samples, and hares in 14 samples, while grouse and deer were rare. The relative frequency of occurrence analysis showed identical rankings for prey and not much percentage difference, largely because most samples consisted of only one prey item. The biomass results show red squirrels (54.0%) and small mammals (24.6%) dominated the winter diet of bobcats.

**Table 2:** The winter diet of bobcats in northwest Montana

	Prey (total detections)	AFO (prey/total samples, *N* = 78)	RFO (prey/total detections, *N* = 94)	Biomass (% consumed in total diet)
Deer	5	6.4	5.3	8.5
Snowshoe hare	14	17.9	14.9	12.2
Red squirrel	38	48.7	40.4	54.0
Grouse	10	12.8	10.6	0.7
Cricetid rodents, total	27	34.6	28.7	24.6
Arvicolinae[Link]	10	12.8	10.6	16.5
Neotominae[Link]	10	12.8	10.6	5.6
Unknown Cricetidae[Link]	7	9.0	7.4	2.5

Notes

Scats were collected between December 2009 and April 2010 and between December 2010 and March 2011. Bobcat carcasses were collected in December 2009 and 2010 (*M* = 28; *F* = 19). After exclusion of trap bait and incidentally ingested items, samples totaled 78 (scat = 16; colon = 37; stomachs = 25). Dietary biomass consumed was estimated via a bobcat‐specific regression relating sample biomass to ingested biomass of different prey types (Baker et al., 1993).

One sample was muskrat, *Ondatra zibethicus*, in a female bobcat stomach. All others were voles.

Eight of 10 Neotominae samples were *Neotoma cinerea*. The others were *Peromyscus maniculatus*.

Unknown Cricetidae samples had no diagnostic bones or teeth, but fur indicated a Cricetid.

**Figure 2:**
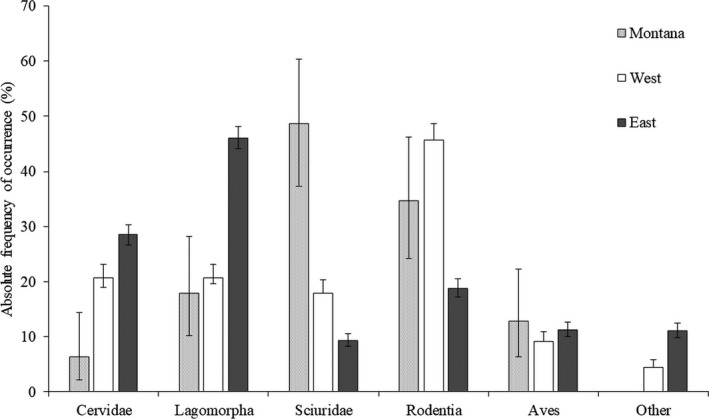
Prey consumed in winter by bobcats in northwest Montana (this study) versus other studies (Table 4). The “Sciuridae” category reflects red squirrel for Montana bobcats, but in other northern latitudes includes Eastern gray squirrel and northern flying squirrel. Error bars are the exact 95% binomial confidence intervals

Across the northern range, bobcats displayed diets that varied substantially, with most of the variation arising from differences between Eastern and Western groups (Table 3). Western bobcats consumed far more squirrels and rodents but fewer lagomorphs than did Eastern bobcats (Figure 2). Bobcats in Eastern locations consumed more lagomorphs and cervids in their diets than did Western bobcats. We located only two studies that addressed bobcat diets from the Great Lakes states (Gilbert, 2000; Rollings, 1945); in this region, bobcats ate primarily deer and snowshoe hares.

**Table 3:** Winter diet of bobcats in the northern United States and southern Canada (1939–2005). These results are based on absolute frequency of occurrence

Location	Sample	*N*	Cervid[Link]	Lagomorph[Link]	Tree squirrel[Link]	Other rodent[Link]	Bird	Other[Link]	References
Western									
MT	Scat, colon, stomach	78	6.4	17.9	48.7	34.6	12.8	0.0	This study
ID	Scat	135	43.8[Link]	1.5	2.2	88.1	3.7	0.0	Koehler and Hornocker (1989)
OR	Scat	499	35.0[Link]	38.0	11.0	32.0	7.0	4.0	Toweill and Anthony (1988)
WA (E)	Stomach	324	7.0	26.0	11.0	48.0	7.0	9.0	Knick, Sweeney, Alldredge, and Brittell (1984)
WA (W)	Stomach	123	11.0	20.0	17.0	26.0	15.0	9.0	Knick et al. (1984)
Average			20.6	20.7	18.0	45.7	9.1	4.4	
Midwestern									
MN	Stomach	50	35.0	44.1	0.9	4.3	1.6	15.4	Rollings (1945)
WI	Stomach	309	45.3	18.4			2.9		Gilbert (2000)[Link]
Average			40.2	31.3			2.3		
Eastern									
ME	Stomach, colon	88	40.9	21.6	14.0	20.0	12.0	21.0	Westfall (1956)
ME	GI tract	230	12.4	50.6	5.0	12.4	12.9	8.8	Litvaitis, Clark, et al. (1986)
ME	Scat	346	29.4[Link]	64.7	0.0	14.7	5.9	2.9	Litvaitis and Harrison (1989)
NS	Stomach	666	17.1	71.0	4.8	16.4	6.6	3.7	Matlack and Evans (1992)
MA, VT, ME, NY	Stomach, colon	208	32.2	60.1	11.5	12.1	5.3	18.8	Pollack (1951)
MA	Scat	250	28.0	52.0	11.2	16.0	3.6	10.4	Pollack (1951)
NH	GI tract	388	22.4	48.9	18.8	11.9	0.0	0.0	Litvaitis et al. (1984)
PA	Stomach	85	42.0	15.0	3.0	21.0	39.0	6.0	McLean, McKay, and Lovallo (2005)
VT	Stomach	140	32.0	31.0	13.0	45.0	16.0	28.0	Hamilton and Hunter (1939)
Average			28.5	46.1	9.0	18.8	11.3	11.1	

Typically deer, but Toweill and Anthony (1988) includes 4% elk, Litvaitis and Harrison (1989) includes 5.9% moose, and Koehler and Hornocker (1989) includes 15.6% bighorn sheep (*Ovis canadensis*) and 1.5% unknown.

Snowshoe hare and *Sylvilagus* spp.

Eastern gray squirrel (*Sciurus carolinensis*), American red squirrel, and northern flying squirrel (*Glaucomys sabrinus*).

This grouping includes voles, mice, ground squirrels, and mountain beaver (*Aplodontia rufa*), and the rare report of ground squirrel spp.; hibernating ground squirrels were not available to bobcats in our Montana study.

Other includes large rodents >2 kg, that is, beaver (*Castor canadensis*), woodchuck (*Marmota monax*), and marmots (*Marmota* spp.). Bobcats also consumed raccoons (*Procyon lotor*), porcupine (*Erethizon dorsatum*), skunks (*Spilogale* and *Mephitis* spp.), and opossum (*Didelphis virginiana*), lynx, bobcat, mink (*Neovison vison*), fox (*Vulpes vulpes*), domestic cat (*Felis catus*), and otter (*Lontra canadensis*). Bobcats also consumed fish, vegetation, and berries.

Gilbert (2000) uses different prey categories and presents results in proportion biomass. We present values for deer, hare, and birds by calculating stomachs with that item present divided by total sample size. Gilbert lumps “medium” and “small” mammals, so we could not separate squirrels from other rodents.

Winter dietary niche breadth of bobcats ranged from a low of 1.03 (standardized = 0.01) in Idaho to 5.58 (standardized = 0.92) in Eastern Washington (Table 4). In the Eastern bobcat populations, winter dietary niche breadth ranged from a low of 1.76 (standardized 0.15) in Nova Scotia where bobcats consumed high proportions of snowshoe hares (71% of winter diet) to a high of 3.19 in a 1986 Maine study, where bobcats still consumed over 50% of their winter diet as hares and cottontails. However, bobcat niche breadth among regions was very similar, and indicated generalized diet although the prey composition of regional diets was highly variable.

**Table 4:** Niche breadths of bobcat diets in northern locations within their geographic range

Location	Niche breadth	Standardized niche breadth
Western		
Idaho	1.03	0.01
Montana	2.44	0.29
Oregon	2.58	0.32
Eastern Washington	5.58	0.92
Western Washington	3.05	0.41
Average	2.94	0.39
Eastern		
Maine (Westfall, 1956)	3.01	0.40
Maine (Litvaitis, Clark, et al., 1986; Litvaitis, Sherburne, et al., 1986)	3.19	0.44
Maine (Litvaitis & Harrison, 1989)	1.88	0.18
Nova Scotia	1.76	0.15
New England (Pollack, 1951)	1.88	0.15
MA	2.51	0.30
New Hampshire	2.95	0.39
Pennsylvania	2.50	0.30
Vermont	1.92	0.18
Average	2.40	0.28

Dietary overlap was variable in Western bobcat populations (Table 5), ranging from 55.7% to 91.5%, but dietary overlap was significant overall (observed $\overset{-}{x}$ = 77.1%, variance = 0.013, *p* = 0.003). Bobcats in the Eastern region also shared significant dietary overlap (observed $\overset{-}{x}$ = 81.6%, variance = 0.022, *p* < 0.001), with a range of 50.8%–99.2%. Winter diets of bobcats differed significantly between these broad regions (Figure 2; *G* = 28.24, *df* = 5, *p* < 0.001).

**Table 5:** Pairwise comparison of bobcat populations within broad regions (Western and Eastern) using EcoSim 7.72 (Entsminger, 2014) to calculate niche using Pianka's (1974) index

(a) Western region					
Percent overlap					
	Idaho	Montana	Oregon	E. WA	W. WA
Idaho		0.557	0.727	0.694	0.820
Montana			0.662	0.867	0.763
Oregon				0.874	0.834
E. WA					0.915

(b) Eastern region									
Percent overlap									
	Maine	Maine	Maine	Nova Scotia	New England	MA	NH	PA	VT
Maine		0.700	0.721	0.641	0.828	0.826	0.738	0.850	0.925
Maine			0.961	0.983	0.951	0.953	0.918	0.596	0.749
Maine				0.981	0.960	0.972	0.942	0.600	0.713
Nova Scotia					0.942	0.955	0.944	0.508	0.687
New England						0.992	0.948	0.621	0.798
MA							0.971	0.633	0.816
NH								0.538	0.724
PA									0.781

Montana bobcats show the greatest dietary similarity with Eastern Washington bobcats; however, Western and Eastern bobcat populations have diets that are significantly different (Figure 2; *G* = 28.24, *df* = 5, *p* < 0.01).

## 4 DISCUSSION

Bobcats in northwest Montana did not consume many snowshoe hares. Instead, bobcats primarily ate red squirrels and small mammals, as revealed by absolute frequency of occurrence (48.7% and 34.6%, respectively), relative frequency (40.4% and 28.7%), and the regression‐based estimates of biomass (54.0% squirrel and 24.6% small mammal). The ~1.4‐kg snowshoe hare was not eaten much despite being abundant; average hare densities in summers 2009 and 2010 at eight live‐trapping sites located on the northern half of the study area were ~0.9 hares/ha (Mills and Hodges, unpubl.). This average density is much higher than hare densities reported in Glacier National Park to the east (Cheng, Hodges, & Mills, 2015), central Washington (Lewis, Hodges, Koehler, & Mills, 2011), or Wyoming (Hodges, Mills, & Murphy, 2009); bobcats presumably could have eaten far more hares than they did.

Although we would need prey densities for the entire prey base to definitively address how much of the bobcat diet in northwestern Montana reflects prey abundance versus bobcat prey selection, we do find it striking that so few hares were consumed in the face of comparatively high hare abundances. Bobcats clearly used the entire local prey base, but with a focus on small mammals and red squirrels. It is not clear whether bobcats found it easier to hunt these species rather than snowshoe hares in deep snow. The studies showing bobcats consuming snowshoe hares in higher proportions are from Eastern populations, in regions that are typically flatter and with lower elevations, and thus are without the deep, persistent snows that bobcats in this Montana study area experienced. Deer would be available to Eastern bobcats throughout the winter, but in Western localities, deer often overwinter in lowland valleys rather than in forested uplands, thus becoming less available for bobcats that overwinter at higher elevations. The predator communities also differ, with many Western forests supporting top predators such as wolves (*Canis lupus*) and mountain lions (*Puma concolor*), whereas Eastern forests often lack these species. Although the top predators do not often consume snowshoe hares, their presence could alter the behavior of bobcats. Finally, recent genetic evidence points to deep lineage splits between Western and Eastern bobcats (Croteau, Heist, Nielsen, Hutchinson, & Hellgren, 2012; Reding, Bronikowski, Johnson, & Clark, 2012), so there could potentially be subtle behavioral or phenotypic variation between these groups as well that would affect hunting success of prey selection.

### 4.1 Do Montana bobcats have more specialized winter diets than other bobcats?

Montana bobcats ate significantly more squirrels and small mammals and fewer snowshoe hares than did bobcats from other northern forests. Such dependence on rodents is more similar to bobcat diets across their southern range (McCord & Cardoza, 1982; Anderson, 1987; Rolley, 1987; Larivière and Walton 1997; Tewes, Mock, & Young, 2002), but is atypical compared to bobcats in other northern forests. Squirrels and Cricetid rodents combined comprised on average ~41% of bobcat diet in 12 studies from northern latitudes, yet winter diet of bobcats in Montana and in Idaho (Koehler & Hornocker, 1989) was dominated by rodents (~83% and ~90%, respectively), likely due to similarities in regional topography, vegetation, climate, and prey types.

Bobcats in Montana show a standardized niche breadth of 0.29, whereas bobcats in Western populations show a broader standardized niche breadth of 0.39. Although Montana bobcats did show statistically significant dietary overlap with other bobcats in the Western region, their lower standardized niche breadth points toward semi‐specialization on red squirrels and other rodents in Montana. Standardized niche breadth for all winter bobcat diets was 0.32 (range: 0.01–0.92); these values are slightly lower than the values for other cat species considered to be generalist predators, including mountain lions in Brazil (0.47; de Azevedo, 2008) and Peru (0.49; Emmons, 1987); jaguar (*Panthera onca*) in Brazil (0.41; de Azevedo, 2008); and pampas cats (*Leopardus pajeros*, 0.44) and Geoffroy's cats (*Leopardus geoffroyi*, 0.52; Berg, 2007). These studies of felid species’ dietary niche breadth take place in warmer, tropical climates, and the slightly lower winter niche breadth of northern bobcats could simply reflect paucity of prey species available during winter months in these areas, as many potential prey species hibernate or migrate seasonally.

Other studies of niche breadth of bobcats have used the nonstandardized niche breadth measure; for example, winter diet of bobcats in California had *B* = 8.97 (Neale & Sacks, 2001). Bobcats in this southern locale had a much higher nonstandardized niche breadth than bobcats in Montana (*B* = 2.44) or bobcats in other northern latitudes (*B* = 2.59). This comparison reflects the fact that southern bobcat population have more prey species available to them in winter and make use of this broader suite of prey.

We do note that like most studies on bobcat diet, we had to lump some prey together (grouse spp., small rodents, even two deer species) because the gut or scat samples were too degraded to be confident of species identity within these groups. Had we been able to identify all prey remains to species, the total number of prey species consumed would be higher and the percentage of diet composed of each species would be lower for these mingled groups. Our estimate of snowshoe hares and red squirrels in the diet would remain the same for absolute frequency of occurrence and biomass, but would drop for relative frequency if smaller prey species had been separated out. When we compare our results to those from other studies, these problems continue, and in some studies, bobcats also ate other lagomorphs. We also note our survey draws together literature from 1945 to present, and given the large changes in climate and land use over this time span, we suspect there is almost certainly large temporal variation of bobcat diets within each region as well. Despite these challenges, bobcats show clear regional differences in winter diets across their northern range, as exemplified by the range in lagomorphs consumed, which ranged from 1.5% to >90% of the diet.

### 4.2 Is there evidence for dietary competition between lynx and bobcats?

Our data are consistent with dietary partitioning between bobcats and lynx. In the Yukon, Washington, British Columbia, Alberta, and Montana, lynx consumed 50%–82% snowshoe hare and 13%–35% red squirrel (Apps, 2000; Aubry, Koehler, & Squires, 2000; O'Donoghue et al., 1998; Squires & Ruggiero, 2007). Even in Colorado, in the southern range margin for lynx, lynx ate 65%–98% snowshoe hares, with red squirrels as the major secondary prey (Ivan & Shenk, 2016). The bobcat diet in Montana consisted of 49% red squirrels and 18% snowshoe hares (AFO); along the northern range margin, bobcats consumed 1.5%–71% hares and 0%–49% red squirrels. Across these northern regions, bobcats also consumed a wide range of other prey, including deer, many species of small mammal, and birds.

Thus, lynx and bobcat display fundamentally different dietary strategies: Lynx are snowshoe hare specialists (with red squirrels as major secondary prey) throughout their entire range, regardless of the underlying geography, snow conditions, or prey abundances, whereas bobcats are clearly generalists, with their diets varying substantially across time and space. It remains an open question across the regions where lynx and bobcats co‐occur whether this overlap in prey use is sufficient to affect behavior or population sizes of either predator species. Nor do we know whether the diets of lynx and bobcats reflect active niche separation or simply reflect different hunting abilities in deep snow. It would also be useful to examine summer bobcat diets in their northern range, to see how varied these diets are in relation to winter diets and in comparison with lynx.

In another congeneric pair, Lovari et al. (2013) report that snow leopards (*Panthera uncia*) and common leopards (*Panthera pardus*) used different habitats, but these species showed much higher dietary overlap than we found here. Felid predators have a large suite of behaviors (scent‐marking, timing of movements, diet, and habitat selection, inter alia) that are likely employed in areas of sympatry to reduce harmful direct interactions with members of other species (Ramesh, Kalle, Sankar, & Qureshi, 2012). Here, we seem to see dietary separation, and some studies have hinted at fine‐scale spatial separation among these species as well (Gooliaff et al., 2018; Scully, Fisher, Miller, & Thornton, 2018).

These dietary results suggest that bobcats and lynx may not compete in the northern forests where they are sympatric, based on the relatively low abundance of hares in the bobcat diets that we observed. Large‐scale occupancy models have inferred competition from patterns of overlap of the two species (Peers, Thornton, & Murray, 2013; Scully et al., 2018), but detailed behavioral information (e.g. from radio‐collared individuals of both species) is lacking, as is information showing any demographic impacts of one species on the other in areas where they are sympatric. The recent Species Status Assessment for lynx (US Fish & Wildlife Service, 2017) highlights that a key uncertainty in lynx ecology is the extent to which they compete with bobcats, other mesocarnivores, and even raptors and owls.

Detailed radiotelemetry work would help resolve how much spatial overlap lynx and bobcats tolerate. More information on prey availabilities would also help resolve how limiting prey are for either species; when hares are at low density, even a small amount of predation on hares or red squirrels by bobcats could be damaging to lynx populations. Overall, we conclude that even under environmental conditions that likely favor specialists, bobcats retain a flexible diet and thus are enabled to persist in difficult winter conditions in their northern range.

## AUTHORS’ CONTRIBUTIONS

RN and KH jointly developed the idea and study design and cowrote the manuscript. RN conducted the fieldwork, autopsies, scat analyses, and statistics. KH obtained most of the funding for this work.

## DATA ACCESSIBILITY

Upon acceptance for publication, the authors will make data publicly available. Sampling locations, bobcat samples (stomachs, colons, and scats), prey species present in each sample, and sample dry weights are archived with FigShare (https://dx.doi.org/10.6084/m9.figshare.7068680).

## References

[ece34576-bib-0001] Ackerman, B. B. , Lindzey, F. G. , & Hemker, T. P. (1984). Cougar food habits in southern Utah. Journal of Wildlife Management, 48, 147–155. 10.2307/3808462

[ece34576-bib-0002] Anderson, E. M. (1987). Bobcat predation on a red‐tailed hawk. Southwestern Naturalist, 32, 149–150. 10.2307/3672027

[ece34576-bib-0003] Apps, C. (2000). Space‐use, diet, demographics, and topographic associations of lynx in the southern Canadian Rockies In Ruggiero L. F., Aubry K. B., Buskirk S. W., Koehler G. M., Krebs C. J., McKelvey K. S., & Squires J. R. (Eds.), Ecology and conservation of lynx in the United States (pp. 351–372). Denver, CO: University of Colorado Press.

[ece34576-bib-0004] Aubry, K. B. , Koehler, G. M. , & Squires, J. R. (2000). Ecology of Canada lynx in southern boreal forests In Ruggiero L. F., Aubry K. B., Buskirk S. W., Koehler G. M., Krebs C. J., McKelvey K. S., & Squires J. R. (Eds.), Ecology and conservation of lynx in the United States (pp. 373–396). Denver, CO: University of Colorado Press.

[ece34576-bib-0005] Bailey, T. N. (1974). Social organization in a bobcat population. Journal of Wildlife Management, 38, 435–446. 10.2307/3800874

[ece34576-bib-0006] Baker, L. A. , Warren, R. J. , & James, W. E. (1993). Bobcat prey digestibility and representation in scats. Proceedings of the Annual Conference of the Southeast Association of Fish and Wildlife Agencies, 47, 71–79.

[ece34576-bib-0007] Berg, J. E. (2007). The carnivore assemblage of La Payunia reserve, Patagonia, Argentina: Dietary niche, prey availability, and selection. M.Sc. thesis. Missoula, MT: University of Montana.

[ece34576-bib-0008] Buskirk, S. W. , Ruggiero, L. F. , & Krebs, C. J. (2000). Habitat fragmentation and interspecific competition: Implications for lynx conservation In Ruggiero L. F., Aubry K. B., Buskirk S. W., Koehler G. M., Krebs C. J., McKelvey K. S., & Squires J. R. (Eds.), Ecology and conservation of lynx in the United States (pp. 83–100). Denver, CO: University of Colorado Press.

[ece34576-bib-0009] Cheng, E. , Hodges, K. E. , & Mills, L. S. (2015). Impacts of fire on snowshoe hares in Glacier National Park, Montana. Fire Ecology, 11, 119–136.

[ece34576-bib-0010] Croteau, E. K. , Heist, E. J. , Nielsen, C. K. , Hutchinson, J. R. , & Hellgren, E. C. (2012). Microsatellites and mitochondrial DNA reveal regional population structure in bobcats (*Lynx rufus*) of North America. Conservation Genetics, 13, 1637–1651. 10.1007/s10592-012-0416-0

[ece34576-bib-0011] de Azevedo, F. C. C. (2008). Food habits and livestock depredation of sympatric jaguars and pumas in the Iguaçu National Park Area, South Brazil. Biotropica, 40, 494–500. 10.1111/j.1744-7429.2008.00404.x

[ece34576-bib-0012] Emmons, L. H. (1987). Comparative feeding ecology of felids in a Neotropical rainforest. Behavioral Ecology and Sociobiology, 20, 271–283. 10.1007/BF00292180

[ece34576-bib-0013] Entsminger, G. L. (2014). EcoSim professional: Null modeling software for ecologists, Version 1. Montrose, CO: Acquired Intelligence Inc., Kesey‐Bear, and Pinyon Publishing Retrieved from https://www.garyentsminger.com/ecosim/index.htm

[ece34576-bib-0014] Flathead National Forest (2006). Proposed land management plan, appendix 58, flathead forest plan amendment 21, appendix IV, Subbasins and geographic unit descriptions.

[ece34576-bib-0015] Foresman, K. R. (2001). The Wild Mammals of Montana. Special Publication No. 12. American Society of Mammalogists.

[ece34576-bib-0016] Fuller, T. K. , Berg, W. E. , & Kuehn, D. W. (1985). Bobcat home range size and daytime cover‐type use in northcentral Minnesota. Journal of Mammalogy, 66, 568–571. 10.2307/1380938

[ece34576-bib-0017] Gilbert, J. W. (2000). Impacts of reestablished fishers on bobcat populations in Wisconsin. Ph.D. Dissertation. Madison, WI: University of Wisconsin.

[ece34576-bib-0018] Gooliaff, T. J. , & Hodges, K. E. (2018). Historical distributions of bobcats and Canada lynx suggest no range shifts in British Columbia. Canadian Journal of Zoology. 10.1139/cjz-2018-0010

[ece34576-bib-0019] Gooliaff, T. J. , Weir, R. D. , & Hodges, K. E. (2018). Estimating bobcat and Canada lynx distributions in British Columbia. Journal of Wildlife Management, 82, 810–820. 10.1002/jwmg.21437

[ece34576-bib-0020] Hamilton, W. J. Jr , & Hunter, R. P. (1939). Fall and winter food habits of Vermont bobcats. Journal of Wildlife Management, 3, 99–103. 10.2307/3796351

[ece34576-bib-0021] Hodges, K. E. , Mills, L. S. , & Murphy, K. M. (2009). Distribution and abundance of snowshoe hares in Yellowstone National Park. Journal of Mammalogy, 90, 870–878. 10.1644/08-MAMM-A-303.1

[ece34576-bib-0022] Hurlbert, S. H. (1978). The measurement of niche overlap and some relatives. Ecology, 59, 67–77. 10.2307/1936632

[ece34576-bib-0023] Ivan, J. S. , & Shenk, T. M. (2016). Winter diet and hunting success of Canada lynx in Colorado. Journal of Wildlife Management, 80, 1049–1058. 10.1002/jwmg.21101

[ece34576-bib-0024] Knick, S. T. , Sweeney, S. J. , Alldredge, J. R. , & Brittell, J. D. (1984). Autumn and winter food habits of bobcats in Washington State. Great Basin Naturalist, 44, 70–74.

[ece34576-bib-0025] Koehler, G. M. , & Hornocker, M. G. (1989). Influences of season on bobcats in Idaho. Journal of Wildlife Management, 53, 197–202.

[ece34576-bib-0026] Krebs, C. J. (1998). Ecological methodology. San Francisco, CA: Benjamin Cummings.

[ece34576-bib-0027] Larivière, S. , & Walton, L. R. (1997). Lynx rufus. Mammalian Species, 563, 1–8.

[ece34576-bib-0028] Levins, R. (1968). Evolution in changing environments: some theoretical explorations (p. 120). Princeton, New Jersey: Princeton University Press.

[ece34576-bib-0029] Lewis, C. W. , Hodges, K. E. , Koehler, G. M. , & Mills, L. S. (2011). Influence of stand and landscape features on snowshoe hare abundance in fragmented forests. Journal of Mammalogy, 92, 561–567. 10.1644/10-MAMM-A-095.1

[ece34576-bib-0030] Litvaitis, J. A. , Clark, A. G. , & Hunt, J. H. (1986). Prey selection and fat deposits of bobcats (*Felis rufus*) during autumn and winter in Maine. Journal of Mammalogy, 67, 389–392.

[ece34576-bib-0031] Litvaitis, J. A. , & Harrison, D. J. (1989). Bobcat‐coyote niche relationships during a period of coyote population increase. Canadian Journal of Zoology, 67, 1180–1188. 10.1139/z89-170

[ece34576-bib-0032] Litvaitis, J. A. , Sherburne, J. A. , & Bissonette, J. A. (1986). Bobcat habitat use and home range size in relation to prey density. Journal of Wildlife Management, 50, 110–117.

[ece34576-bib-0033] Litvaitis, J. A. , Stevens, C. L. , & Mautz, W. W. (1984). Age, sex, and weight of bobcats in relation to winter diet. Journal of Wildlife Management, 48, 632–635. 10.2307/3801206

[ece34576-bib-0034] Lovari, S. , Minder, I. , Ferretti, F. , Mucci, N. , Randi, E. , & Pellizzi, B. (2013). Common and snow leopards share prey, but not habitats: Competition avoidance by large predators? Journal of Zoology, 291, 127–135.

[ece34576-bib-0035] Malo, A. F. , Lozano, J. , Huertas, D. L. , & Virgós, E. (2004). A change of diet from rodents to rabbits (*Oryctolagus cuniculus*): Is the wildcat (*Felis silvestris*) a specialist predator? Journal of Zoology, 263, 401–407. 10.1017/S0952836904005448

[ece34576-bib-0036] Matlack, C. R. , & Evans, A. J. (1992). Diet and condition of bobcats (*Lynx rufu*s) in Nova Scotia during autumn and winter. Canadian Journal of Zoology, 70, 1114–1119.

[ece34576-bib-0037] McCord, C. M. (1974). Selection of winter habitat by bobcats (*Lynx rufus*) on the Quabbin Reservation, Massachusetts. Journal of Mammalogy, 55, 428–437. 10.2307/1379010

[ece34576-bib-0038] McCord, C. M. , & Cardoza, J. E. (1982). Bobcat and lynx: *Felis rufus* and* F. lynx* In Chapman J. A. & Feldhamer G. A.(Eds.), Wild mammals of North America: Biology, management, and economics (pp. 728–768). Baltimore, MD: Johns Hopkins University Press.

[ece34576-bib-0039] McLean, M. L. , McKay, T. S. , & Lovallo, M. J. (2005). Influence of age, sex and time of year on diet of the bobcat (*Lynx rufus*) in Pennsylvania. American Midland Naturalist, 153, 450–453. 10.1674/0003-0031(2005)153[0450:IOASAT]2.0.CO;2

[ece34576-bib-0040] Moore, T. D. , Spence, L. E. , & Dugnolle, C. E. (1974). Identification of the dorsal guard hairs of some mammals of Wyoming. Wyoming Game and Fish Department Bulletin No. 14.

[ece34576-bib-0041] Mowat, G. , Poole, K. G. , & O’Donoghue, M. (2000). Ecology of lynx in northern Canada and Alaska In Ruggiero L. F., Aubry K. B., Buskirk S. W., Koehler G. M., Krebs C. J., McKelvey K. S., & Squires J. R. (Eds.), Ecology and conservation of lynx in the United States (pp. 265–306). Denver, CO: University of Colorado Press.

[ece34576-bib-0042] Murray, D. L. , & Boutin, S. (1991). The influence of snow on lynx and coyote movements: Does morphology affect behavior? Oecologia, 88, 463–469. 10.1007/BF00317707 28312614

[ece34576-bib-0043] Neale, J. C. C. , & Sacks, B. N. (2001). Resource utilization and interspecific relations of sympatric bobcats and coyotes. Oikos, 94, 236–249. 10.1034/j.1600-0706.2001.940204.x

[ece34576-bib-0044] Newbury, R. K. (2013). Behavioral ecology of the bobcat in a region with deep winter snows (p. 193). Ph.D. Dissertation. University of British Columbia Okanagan.

[ece34576-bib-0045] NOAA [National Oceanic and Atmospheric Administration]. (2017). Daily and monthly temperatures, precipitation, snowfall, and wind speeds. Retrieved from https://www.nws.noaa.gov/climate/index.php?wfo=mso

[ece34576-bib-0046] O’Donoghue, M. , Boutin, S. , Krebs, C. J. , Murray, D. L. , & Hofer, E. J. (1998). Behavioural responses of coyotes and lynx to the snowshoe hare cycle. Oikos, 82, 169–183. 10.2307/3546927

[ece34576-bib-0047] Parker, G. R. , & Smith, G. E. J. (1983). Sex‐ and age‐specific reproductive and physical parameters of the bobcat (*Lynx rufus*) on Cape Breton Island, Nova Scotia. Canadian Journal of Zoology, 61, 1771–1782.

[ece34576-bib-0048] Peers, M. J. L. , Thornton, D. H. , & Murray, D. L. (2013). Evidence for large‐scale effects of competition: Niche displacement in Canada lynx and bobcat. Proceedings of the Royal Society. B, Biological Sciences, 280, 20132495. 24174116 10.1098/rspb.2013.2495 PMC3826238

[ece34576-bib-0049] Pianka, E. R. (1974). Evolutionary ecology. New York, NY: Harper and Row.

[ece34576-bib-0050] Pollack, E. M. (1951). Food habits of bobcats in the New England states. Journal of Wildlife Management, 15, 209–213.

[ece34576-bib-0051] Ramesh, T. , Kalle, R. , Sankar, K. , & Qureshi, Q. (2012). Spatio‐temporal partitioning among large carnivores in relation to major prey species in Western Ghats. Journal of Zoology, 287, 269–275. 10.1111/j.1469-7998.2012.00908.x

[ece34576-bib-0052] Reding, D. M. , Bronikowski, A. M. , Johnson, W. E. , & Clark, W. R. (2012). Pleistocene and ecological effects on continental‐scale genetic differentiation in the bobcat (*Lynx rufus*). Molecular Ecology, 21, 3078–3093. 22548482 10.1111/j.1365-294X.2012.05595.x

[ece34576-bib-0053] Reynolds, J. C. , & Aebischer, N. J. (1991). Comparison and quantification of carnivore diet by faecal analysis: A critique, with recommendations, based on a study of the Fox *Vulpes vulpes* . Mammal Review, 21, 97–122. 10.1111/j.1365-2907.1991.tb00113.x

[ece34576-bib-0054] Rolley, R. E. (1987). Bobcat In Novak M., Baker J. A., Obbard M. E., & Malloch B., (Eds.),Wild furbearer management and conservation in North America (pp. 671–681). Ottawa, ON: Ministry of Natural Resources.

[ece34576-bib-0055] Rollings, C. T. (1945). Habits, foods, and parasites of the bobcat in Minnesota. Journal of Wildlife Management, 9, 131–145. 10.2307/3795892

[ece34576-bib-0056] Roth, J. D. , Marshall, J. D. , Murray, D. L. , Nickerson, D. M. , & Steury, T. D. (2007). Geographical gradients in diet affect population dynamics of Canada lynx. Ecology, 88, 2736–2743. 18051641 10.1890/07-0147.1

[ece34576-bib-0057] Scully, A. E. , Fisher, S. , Miller, D. A. W. , & Thornton, D. H. (2018). Influence of biotic interactions on the distribution of Canada lynx (Lynx canadensis) at the southern edge of their range. Journal of Mammalogy, 99, 760–772. 10.1093/jmammal/gyy053

[ece34576-bib-0058] Sikes, R. S. , & the Animal Care and Use Committee of the American Society of Mammalogists (2016). 2016 Guidelines of the American Society of Mammalogists for the use of wild mammals in research and education. Journal of Mammalogy, 97, 663–688. 10.1093/jmammal/gyw078 29692469 PMC5909806

[ece34576-bib-0059] Squires, J. R. , & Ruggiero, L. F. (2007). Winter prey selection of Canada lynx in northwestern Montana. Journal of Wildlife Management, 71, 310–315. 10.2193/2005-445

[ece34576-bib-0060] Tewes, M. E. , Mock, J. M. , & Young, J. H. (2002). Bobcat predation on quail, birds, and mesomammals In DeMaso S. J., Kuvlesky W. P., Hernandez F., & Berger M. E., (Ed.) Quail V: Proceedings of the Fifth National Quail Symposium (pp. 65–70). Austin, TX: Parks and Wildlife Department.

[ece34576-bib-0061] Toweill, D. E. , & Anthony, R. G. (1988). Annual diet of bobcats in Oregon’s Cascade Range. Northwest Science, 62, 99–103.

[ece34576-bib-0062] Tuomainen, U. , & Candolin, U. (2011). Behavioural responses to human‐induced environmental change. Biological Review, 86, 640–657. 10.1111/j.1469-185X.2010.00164.x 20977599

[ece34576-bib-0063] US Fish and Wildlife Service (2017). Species status assessment for the Canada lynx (Lynx canadensis) Contiguous United States Distinct Population Segment. Version 1.0, October 2017. Lakewood, CO.

[ece34576-bib-0064] Westfall, C. Z. (1956). Foods eaten by bobcats in Maine. Journal of Wildlife Management, 20, 199–200. 10.2307/3797431

[ece34576-bib-0065] Wright, B. E. (2010). Use of chi‐square tests to analyze scat‐derived diet composition data. Marine Mammal Science, 26, 395–401. 10.1111/j.1748-7692.2009.00308.x

